# New Appliances and Things Medical

**Published:** 1908-09-26

**Authors:** 


### NEW APPLIANCES AND THINGS MEDICAL.

### SCHACHT'S DIGESTIVE FLUIDS.

We have received from Messrs. Giles, Schacht and Co., Clifton, Bristol, samples of *Pepsina Liquida c̄ Bismutho* (*Schacht*) and of "*Bisedia*," formerly known as *Liquor Bismuthi Sedativus* (*Schacht*).

The former preparation, *Pepsina Liquida c̄ Bismutho*, is an elegant combination of two well-known products of the firm of Giles, Schacht and Co. Each drachm of this fluid contains a full dose in concentrated form of Schacht's Liquor Bismuthi, together with Schacht's Pepsina Liquida. The bismuth solution was introduced by a member of this firm in 1855, and was the first liquid preparation of bismuth to be placed on the market. It has gained an established position in the eyes of the medical profession for its purity and uniform composition. It is carefully freed from arsenic, silver, copper, and antimony salts and from other contaminations. The liquid pepsine is a clear, active, and stable solution of gastric juice from the pig, and is carefully standardised according to the B.P. tests of peptic activity. It is an excellent and agreeable preparation of pepsin, and we have frequently found it of value in suitable cases of dyspepsia. In the present combination its range of usefulness is distinctly enlarged. It has particular value in the treatment of gastric irritation and in the preliminary treatment of chronic dyspepsia.

"*Bisedia*" is the new name for a liquid combination of Schacht's Liquor Bismuthi and Pepsina Liquida, together with sedatives. Each drachm contains, in a concentrated form, Liquor bismuthi ʒj., pepsina liquida ʒj., morphinæ hydrochlor. gr. $\frac{1}{24}$, acid. hydrocyan. dil. B.P. ♏ ij., and tinct. nucis vomic. ♏ v. This preparation is of considerable service in gastric disease. Its sedative properties are well marked, and we have found acute gastric pain and vomiting rapidly relieved by it. It is a bright clear liquid of a pink colour, without sediment, and perfectly miscible with water. It is quite palatable. In acute catarrhal gastritis it is often very effectual, the more distressing symptoms soon yielding as a rule to the combination of sedatives. Under its former name this preparation was favourably known to many medical practitioners, and its popularity is likely to increase in the future, in spite of the change of title. Both of these preparations may be obtained of wholesale druggists, or will be sent direct by the manufacturers.

### A NEW HOSPITAL FLOOR POLISH.

After ten years' experience in the sanitary treatment of hospital floors and furniture, Mr. E. G. Bawtree has recently introduced a finely concentrated floor polish of high quality which is remarkably moderate in price. In the "Tree" Floor Polish a white-wax basis has been made use of, and the result of trials of this preparation has been most satisfactory in every respect. This polish thoroughly cleanses the floor, and produces a hard brilliant surface, very durable if kept free from grit, etc., by sweeping and a daily dry rubbing. Delivery is made in 14 lb. tins, the price being 9*d*. per lb. The polish is also put up in 1 lb. tins to meet the requirements of many hospital authorities who find this the most convenient way of serving out floor polish. Although lower in price than other brands, this preparation satisfies us that it is their equal in efficiency. The "Tree" germ-proof floor polish is manufactured by Messrs. Bawtree, 9 Fenchurch Buildings, London, E.C.

